# Fulminant Immune Checkpoint Inhibitor–Associated Myocarditis Presenting with Complete Atrioventricular Block After Combined Nivolumab and Ipilimumab Therapy: An Autopsy-Confirmed Case Report

**DOI:** 10.3390/diagnostics16213536

**Published:** 2026-10-28

**Authors:** Jonas Knoblich, Anton Richter, Hamzah Adwan, Thomas J. Vogl, Boris Schmidt, Araba Benyarko, Simon Little, Nihat Firat Sipahi

**Affiliations:** 1 Clinic for Emergency and Intensive Care Medicine, Agaplesion Markus Hospital, Academic Teaching Hospital of the Goethe University, 60431 Frankfurt am Main, Germany; knoblich.jonas@gmail.com (J.K.); anton.richter@web.de (A.R.); simonlittle@web.de (S.L.);; 2 Institute of Radiology, Agaplesion Markus Hospital, Academic Teaching Hospital of the Goethe University, 60431 Frankfurt am Main, Germany; 3 Clinic for Radiology and Nuclear Medicine, Goethe University, University Hospital Frankfurt, 60590 Frankfurt am Main, Germany; t.vogl@em.uni-frankfurt.de; 4 Department of Cardiology, Agaplesion Markus Hospital, Academic Teaching Hospital of the Goethe University, CCB Frankfurt, 60431 Frankfurt am Main, Germany; b.schmidt@ccb.de; 5 OptiPath^®^, MVZ für Pathologie Frankfurt/Main eGbR, 60431 Frankfurt am Main, Germany

**Keywords:** immune checkpoint inhibitor, myocarditis, nivolumab, ipilimumab, renal cell carcinoma, third-degree atrioventricular block, pacemaker

## Abstract

**Background**: Immune checkpoint inhibitor (ICI)-associated myocarditis is a rare but potentially fatal immune-related adverse event that may present with conduction abnormalities, ventricular arrhythmias, cardiogenic shock, and sudden cardiac death. The risk is increased with combined programmed cell death protein 1 (PD-1) and cytotoxic T-lymphocyte-associated protein 4 (CTLA-4) blockade. We report a fatal case of fulminant ICI-associated myocarditis following combined nivolumab and ipilimumab therapy. **Case Presentation**: A 79-year-old man with metastatic clear-cell renal cell carcinoma developed progressive fatigue and exertional dyspnea 14 days after his first and only cycle of nivolumab and ipilimumab. Electrocardiography revealed complete atrioventricular block. High-sensitivity cardiac troponin T increased from 2575 to 11,327 pg/mL, whereas creatine kinase initially decreased from 8690 U/L before rising again. Coronary angiography excluded obstructive coronary artery disease. Temporary transvenous pacing was followed by permanent pacemaker implantation. Despite intensive care, the patient developed progressive hemodynamic deterioration requiring vasopressor support and ultimately fatal cardiorespiratory collapse. ICI-associated myocarditis was not recognized during life; therefore, no myocarditis-directed diagnostic work-up or immunosuppressive treatment was initiated. Post-mortem examination demonstrated pronounced diffuse lymphohistiocytic myocarditis involving both atria and ventricles. ICI-associated myocarditis was identified as the cause of death, with a concomitant retroperitoneal abscess with pancreatic fistulation considered an aggravating condition. **Conclusions**: Complete atrioventricular block accompanied by a marked dynamic troponin increase should raise suspicion of ICI-associated myocarditis, particularly after exclusion of obstructive coronary artery disease. In this case, the diagnosis was established only post mortem. Earlier recognition of this clinical constellation may facilitate timely myocarditis-directed diagnostic evaluation and treatment.

## 1. Introduction

Immune checkpoint inhibitors (ICIs) targeting programmed cell death protein 1 (PD-1), programmed death ligand 1 (PD-L1), and cytotoxic T-lymphocyte-associated protein 4 (CTLA-4) have revolutionized the treatment of a broad spectrum of malignancies and substantially improved survival in patients with advanced cancer [1]. However, by enhancing antitumor immunity, these agents may also trigger immune-related adverse events (irAEs), which can affect virtually every organ system. ICI-associated myocarditis is uncommon, with an incidence of approximately 1% in large clinical series, but carries substantial morbidity and mortality [2,3,4].

Among cardiovascular irAEs, ICI-associated cardiac toxicity presenting, e.g., as myocarditis, is particularly feared because it may rapidly progress to heart failure, conduction abnormalities, malignant ventricular arrhythmias, cardiogenic shock, and sudden cardiac death [2,3,4]. Diagnosis remains challenging, as the initial clinical presentation is frequently nonspecific and may easily be attributed to the underlying malignancy or other treatment-related adverse events. Consequently, early recognition and prompt initiation of immunosuppressive therapy are essential to improve clinical outcomes [5].

The incidence of myocarditis appears to be considerably higher in patients receiving combination immune checkpoint inhibition than in those treated with ICI monotherapy. In particular, dual inhibition of the PD-1 and CTLA-4 pathways with nivolumab and ipilimumab has repeatedly been associated with fulminant myocarditis characterized by rapidly progressive myocardial inflammation, conduction disturbances, malignant ventricular arrhythmias, and a high risk of fatal outcome [6,7]. Increasing awareness of this rare but devastating complication has resulted in growing interest in serial cardiac biomarker assessment and electrocardiographic surveillance for early detection of myocardial involvement [8,9]. Nevertheless, the optimal diagnostic and therapeutic management of ICI-associated myocarditis continues to evolve.

We report the case of a patient with metastatic clear-cell renal cell carcinoma who developed fulminant, autopsy-confirmed ICI-associated myocarditis after combined nivolumab and ipilimumab therapy.

## 2. Case Presentation

A 79-year-old Caucasian man with metastatic clear-cell renal cell carcinoma (UICC stage IV, pT3 pN0 [0/2] pM1 [PUL]) presented to the emergency department with progressive fatigue, reduced exercise tolerance, exertional dyspnea, and intermittent chest discomfort. Symptoms had developed approximately 14 days after the first administration of combined nivolumab and ipilimumab.

The patient had undergone left nephrectomy for clear-cell renal cell carcinoma in December 2025, followed by pulmonary metastasectomy in January 2026. His medical history additionally included prostate cancer treated surgically in December 2025, arterial hypertension, hypercholesterolemia, and chronic kidney disease. His regular medication included candesartan, rosuvastatin, and tamsulosin. There was no known history of clinically manifest cardiac disease. A transthoracic echocardiogram performed on 13 January 2026, before initiation of ICI therapy, demonstrated preserved left ventricular systolic function without relevant regional wall-motion or valvular abnormalities. No reliable pre-treatment hs-cTnT measurement or pre-ICI ECG could be identified in the available retrospective records.

**Figure 1:**
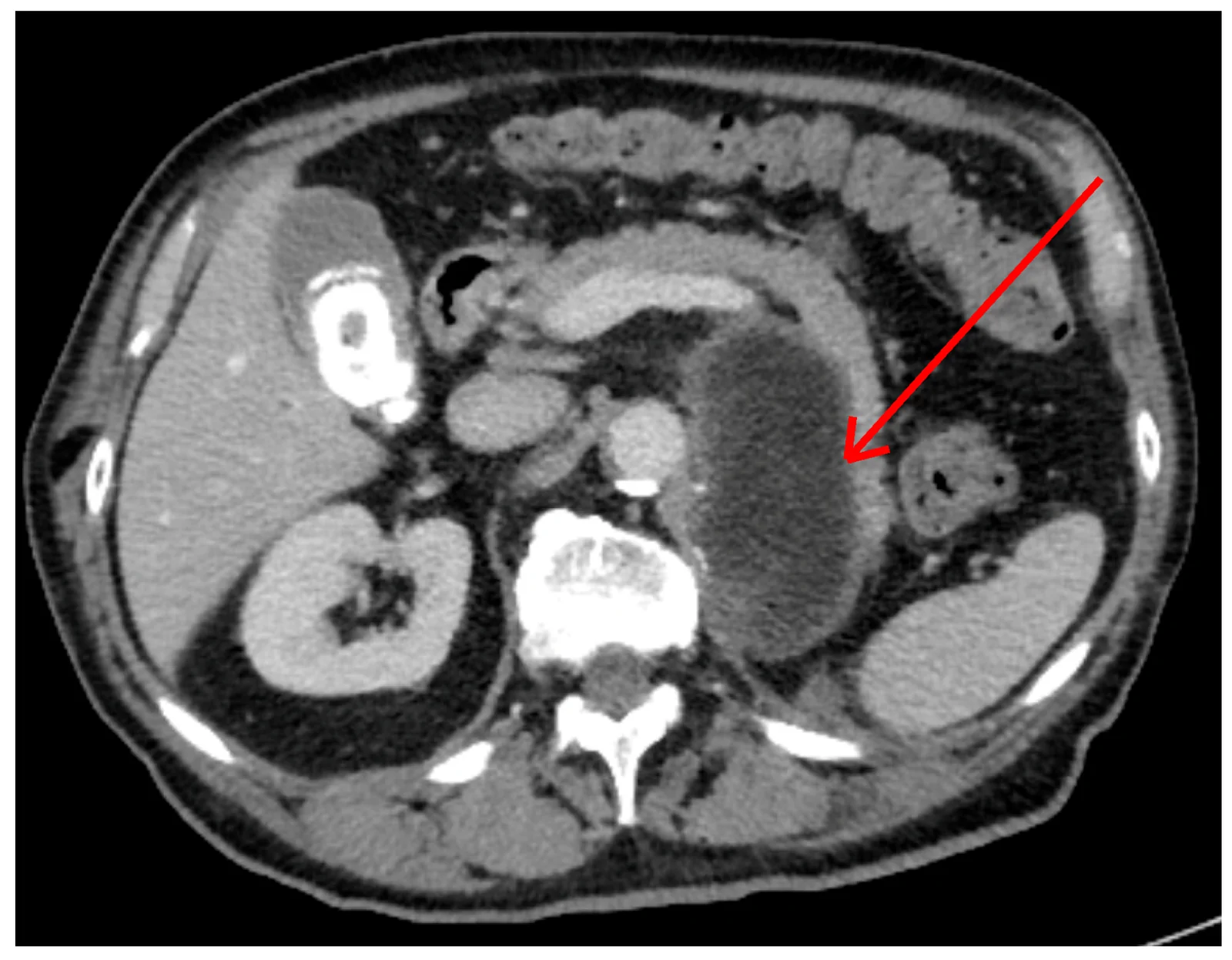
Contrast-enhanced CT performed approximately three weeks before the terminal hospitalization demonstrating a 5.9 × 9.3 cm hypodense mass with peripheral enhancement in the left nephrectomy bed (red arrow), radiologically highly suspicious for a postoperative retroperitoneal abscess with fat necrosis adjacent to the pancreas. The mass was subsequently confirmed at autopsy as an encapsulated retroperitoneal abscess with pancreatic fistulation.

Restaging contrast-enhanced computed tomography performed approximately three weeks before the terminal hospitalization demonstrated mildly progressive bilateral pulmonary metastases and a 5.9 × 9.3 cm hypodense mass with peripheral enhancement in the left nephrectomy bed, radiologically highly suspicious for a postoperative retroperitoneal abscess with fat necrosis adjacent to the pancreas (Figure 1). No pulmonary embolism or pericardial effusion was identified. At that time, the patient was clinically well, and no drainage or other local intervention of the retroperitoneal mass was performed.

Because of metastatic disease, combined immune checkpoint inhibition with nivolumab (3 mg/kg) and ipilimumab (1 mg/kg) was initiated on 17 February 2026. The patient received only one cycle. Approximately 14 days later, he developed the symptoms described above and was admitted on 6 March 2026.

**Figure 2:**
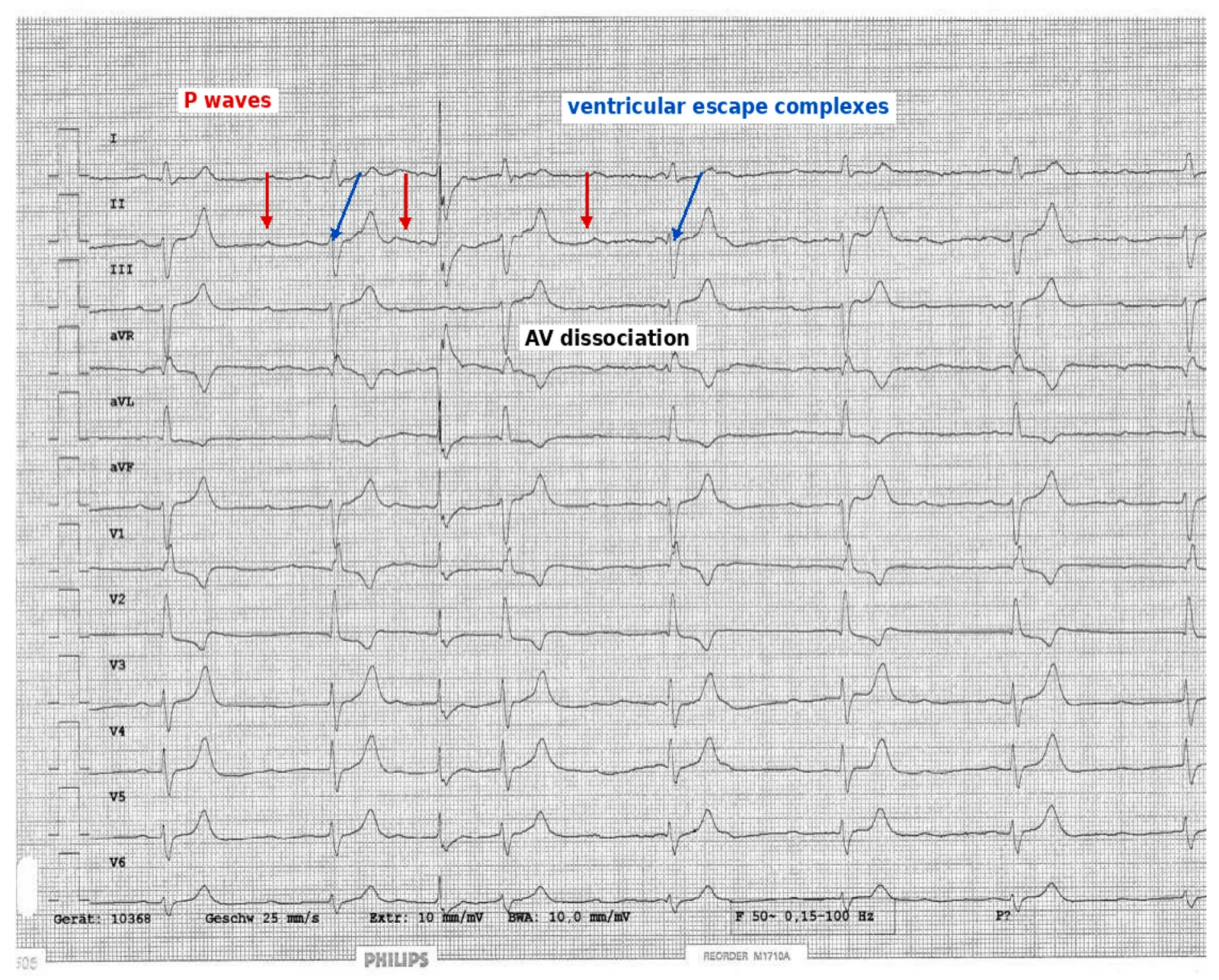
Admission 12-lead electrocardiogram demonstrating complete third-degree atrioventricular block. The contemporaneous ECG report documented a ventricular rate of approximately 35 beats/min and a QRS duration of 140 ms. Red arrows identify representative P waves and blue arrows identify representative ventricular escape complexes, illustrating atrioventricular dissociation.

On admission, electrocardiography demonstrated complete third-degree atrioventricular block with a ventricular rate of approximately 35 beats/min and a QRS duration of 140 ms (Figure 2). T-wave inversion in V1–V2 and aVL was additionally documented. Transthoracic echocardiography demonstrated preserved left ventricular systolic function without regional wall-motion abnormalities or pericardial effusion. High-sensitivity cardiac troponin T (hs-cTnT) was markedly elevated at 2575 pg/mL (reference ≤ 14 pg/mL), increasing to 2857 pg/mL later that evening and 3391 pg/mL the following morning. Creatine kinase (CK) was 8690 U/L (reference ≤ 190 U/L), and CK-MB was 350 U/L (reference ≤ 25 U/L). In the setting of chest discomfort, marked hs-cTnT elevation, and complete AV block, the contemporaneous working diagnoses were non-ST-elevation myocardial infarction (NSTEMI) and third-degree AV block. ICI-associated myocarditis was not considered at this stage. NSTEMI was not confirmed as the final diagnosis, and the elevated troponin concentrations were not specific for an acute coronary syndrome.

**Figure 3:**
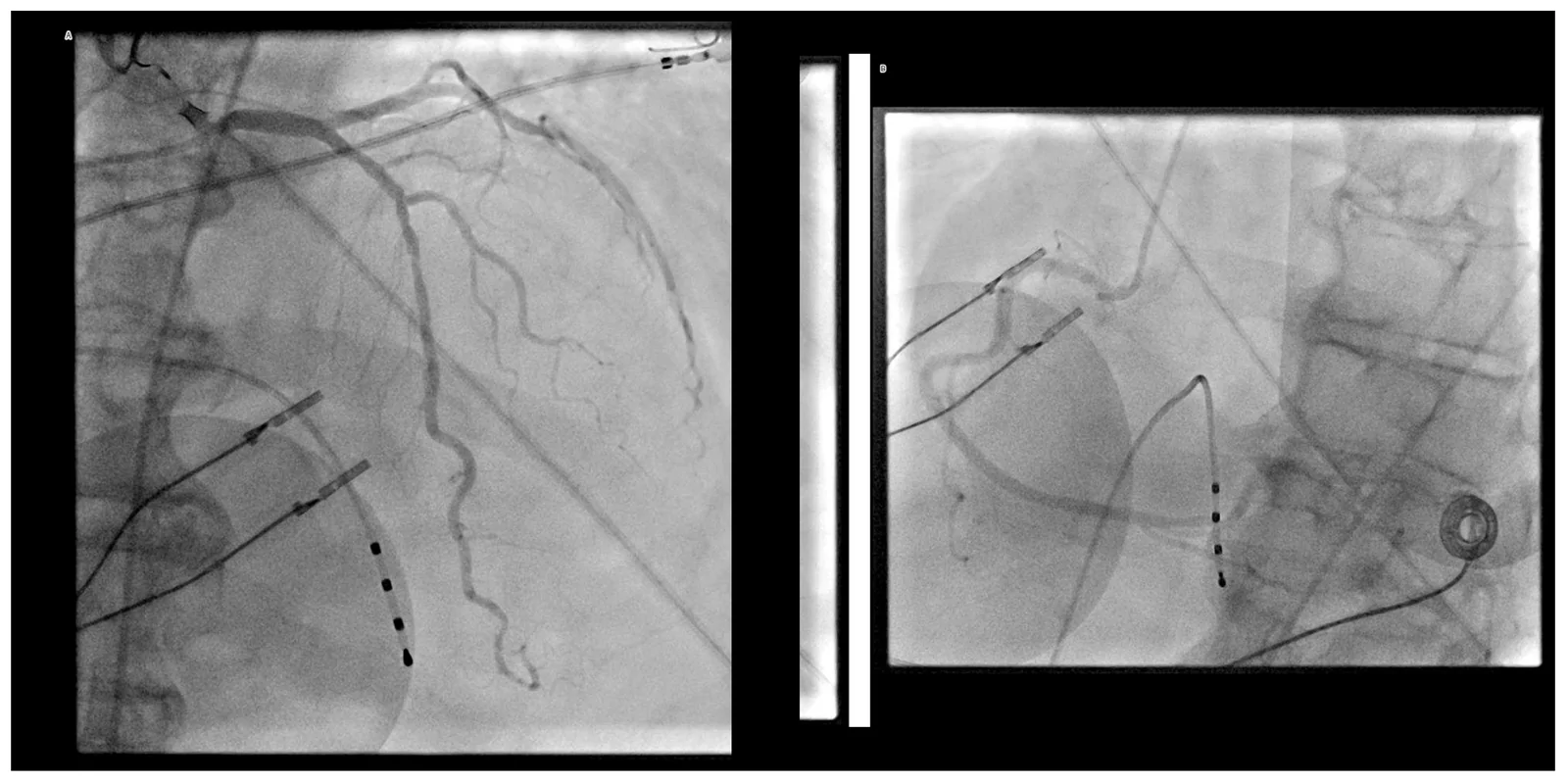
Coronary angiography excluding obstructive coronary artery disease. (**A**) Selective coronary angiographic view demonstrating coronary arteries without obstructive disease. (**B**) Additional angiographic view supporting the absence of obstructive coronary artery disease; the temporary transvenous pacing lead is visible within the right heart. The coronary circulation was right-dominant. Iodinated contrast medium was used; the exact contrast agent could not be reliably retrieved retrospectively.

Coronary angiography was performed on 7 March 2026 for the documented indications of NSTEMI and third-degree AV block and demonstrated a right-dominant coronary circulation without obstructive coronary artery disease (Figure 3). During the same procedure, a temporary transvenous pacing lead was positioned in the right ventricular apex and programmed to VVI 70 beats/min. Because of persistent high-grade atrioventricular conduction disturbance, a permanent dual-chamber pacemaker was implanted on 9 March 2026 and programmed in DDD mode (50–150 beats/min). Subsequent device interrogation documented appropriate pacemaker function with 71% ventricular pacing. The available ECG from 10 March 2026 at 14:06 demonstrated an effective paced rhythm at approximately 70 beats/min; subsequent QRS morphology and repolarization abnormalities therefore have to be interpreted in the context of ventricular pacing.

Despite exclusion of obstructive coronary artery disease, the marked and dynamically increasing hs-cTnT concentration was not recognized as a manifestation of ICI-associated myocarditis. Renal dysfunction was present on admission (creatinine 2.86 mg/dL; eGFR 20 mL/min/1.73 m^2^) and was considered a potential contributor to the elevated absolute hs-cTnT concentration, together with a low-output state associated with complete AV block; after permanent pacemaker implantation, a device-related contribution was also considered. These factors did not explain the pronounced dynamic hs-cTnT increase during hospitalization. ICI-associated myocarditis was therefore not included in the differential diagnosis during the hospitalization. Consequently, cardiac magnetic resonance imaging, myocarditis-directed endomyocardial biopsy, and immunosuppressive treatment were not initiated.

**Figure 4:**
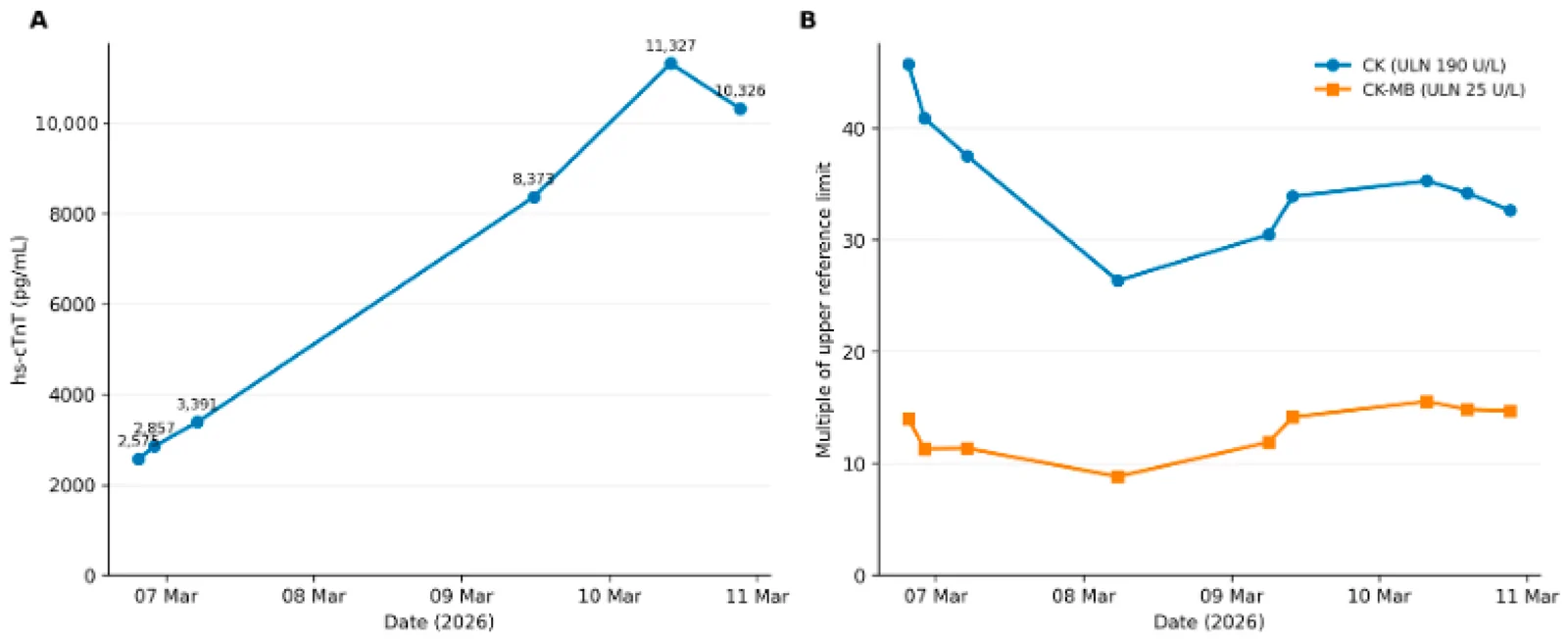
Temporal course of cardiac and muscle injury biomarkers during hospitalization. (**A**) High-sensitivity cardiac troponin T (hs-cTnT) showed a marked dynamic increase, reaching 11,327 pg/mL on 10 March 2026 before decreasing slightly. (**B**) Creatine kinase (CK) and CK-MB, shown relative to their respective upper reference limits, demonstrated a non-monotonic course with an initial decrease followed by a secondary increase. Laboratory upper reference limits were 14 pg/mL for hs-cTnT, 190 U/L for CK, and 25 U/L for CK-MB.

Serial laboratory testing demonstrated progressive myocardial injury. Hs-cTnT increased from 2575 pg/mL on 6 March to 3391 pg/mL on 7 March and 8373 pg/mL on 9 March, reaching 11,327 pg/mL on 10 March before decreasing slightly to 10,326 pg/mL later that day. In contrast, CK showed a non-monotonic course, decreasing from 8690 U/L on admission to 5011 U/L on 8 March before increasing again to 6707 U/L on 10 March. CK-MB showed a similar initial decrease followed by a secondary increase, reaching 389 U/L on 10 March. NT-proBNP was markedly elevated at 20,538 pg/mL on 9 March and increased to 29,534 pg/mL on 10 March (Figure 4).

The marked CK elevation raised the possibility of concomitant ICI-associated myositis. However, specific clinical features of myositis, such as muscle weakness or myalgia, cannot be reliably reconstructed from the available documentation. Because an ICI-associated myositis/myasthenic overlap syndrome was not suspected during the hospitalization, no neurological consultation and no dedicated neuromuscular diagnostic work-up, including AChR/MuSK antibody testing, were performed. Concomitant ICI-associated myositis therefore remained unconfirmed. Rosuvastatin therapy represented an additional potential contributor to the CK elevation.

On 10 March, the patient developed progressive hemodynamic deterioration requiring norepinephrine support at doses of up to 0.5 µg/kg/min. Across the documented serial measurements, C-reactive protein remained moderately elevated (13.5–15.5 mg/L), whereas procalcitonin remained below the laboratory upper reference limit of 0.50 ng/mL (0.36–0.48 ng/mL). Lipase was only minimally elevated, with a maximum of 73 U/L (reference 13–60 U/L), and subsequently decreased to 56 U/L on 10 March. The abdomen remained clinically unremarkable with normal bowel sounds, no upper abdominal pain was documented, and no fever was reported. Thus, there was no clinical or laboratory constellation during the hospitalization suggestive of clinically manifest acute pancreatitis or established systemic sepsis.

In view of the previously known retroperitoneal fluid collection, which had been radiologically described as suspicious for a postoperative abscess, central venous blood cultures were obtained on 10 March, and urine cultures were also obtained during the hospitalization; no tracheal secretion sample was collected. Empirical piperacillin/tazobactam therapy was initiated. All microbiological cultures obtained ultimately remained negative, and no drainage or other source-control procedure was performed. Contemporary clinical documentation described the episode as cardiogenic shock in the setting of complete AV block and prolonged bradycardia. However, this classification cannot be confirmed retrospectively. Repeat bedside echocardiography after pacemaker implantation continued to show preserved global left ventricular systolic function; apical rocking/dyskinesia was noted and considered potentially pacing-related, and the inferior vena cava was described as completely collapsed. Although an infectious contribution could not be excluded, septic shock was not established clinically. The preserved global LV function, evidence of reduced intravascular filling, concomitant retroperitoneal pathology, and later vasopressor requirement preclude definitive retrospective classification of the terminal shock state as purely cardiogenic or purely distributive. The absence of characteristic clinical or laboratory findings did not exclude clinically significant abdominal infection or an infectious contribution to circulatory deterioration.

During the night of 10–11 March 2026, the patient developed progressive hypotension despite volume administration, prompting norepinephrine therapy. Ventricular tachycardia subsequently occurred during central venous catheter placement and was treated with amiodarone 300 mg. At 03:28, the patient became pulseless and cardiopulmonary resuscitation was initiated; the initial documented arrest rhythm was pulseless electrical activity. During ongoing resuscitation, recurrent ventricular tachycardia occurred and required repeated defibrillation. Serial blood-gas analyses documented severe terminal metabolic and circulatory decompensation: at 03:15, arterial pH was 7.228 with lactate 52.0 mg/dL (5.8 mmol/L); during ongoing resuscitation at 04:07, pH had decreased to 7.031 and lactate had increased to 98.6 mg/dL (10.9 mmol/L). Despite prolonged advanced life-support measures, resuscitation was terminated at 04:12 (Table 1). 

Because the rapid fatal deterioration remained insufficiently explained by the clinical diagnoses established during life, ICI-associated myocarditis was considered after death, and an autopsy with targeted histopathological examination of the myocardium was requested to clarify the cause of death.

**Figure 5:**
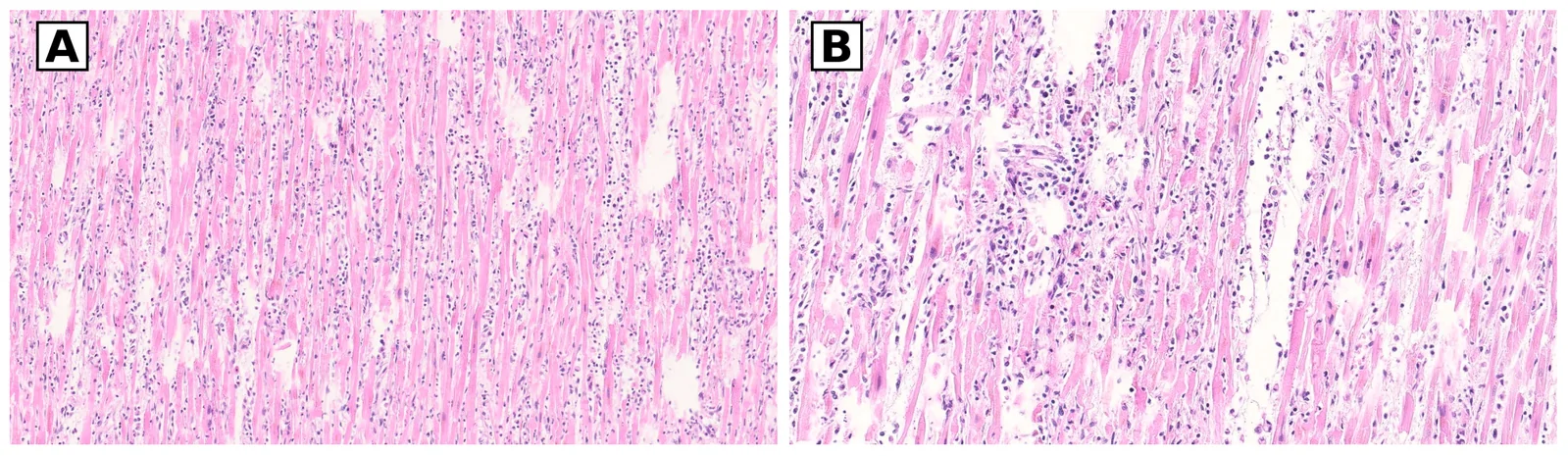
Histopathological findings of ICI-associated myocarditis. Representative hematoxylin and eosin (H&E)-stained myocardial sections obtained at autopsy. (**A**) Representative myocardial section demonstrating lymphohistiocytic inflammatory infiltration. (**B**) Additional representative myocardial section demonstrating lymphohistiocytic myocarditis at a different image scale. Specific objective magnifications and further panel-specific histopathological details cannot be reliably confirmed from the available pathology documentation and should be verified with the reporting pathologist.

Autopsy revealed a heart weight of 500 g and pronounced diffuse lymphohistiocytic myocarditis involving both atria and both ventricles. The pathological assessment identified ICI-associated myocarditis as the cause of death (Figure 5). In addition, an encapsulated retroperitoneal abscess measuring approximately 11 cm × 10 cm × 10 cm was identified in the former left nephrectomy bed, with fistulation to the pancreas and associated necrotizing pancreatitis. Notably, the pancreatic involvement had not been clinically apparent during life, with no upper abdominal pain and no corresponding marked lipase elevation. The abdominal pathology was classified as an aggravating condition rather than the primary cause of death.

## 3. Discussion

ICI-associated myocarditis is an uncommon but potentially fatal immune-related adverse event. Clinical presentation is heterogeneous and may include nonspecific symptoms, elevated cardiac biomarkers, conduction disturbances, ventricular arrhythmias, heart failure, or cardiogenic shock. Combined ICI therapy has been associated with an increased risk of myocarditis compared with ICI monotherapy [3,4,6,7,10]. The present case illustrates the diagnostic challenge of ICI-associated myocarditis presenting predominantly with severe conduction disease and marked myocardial injury shortly after initiation of combined nivolumab and ipilimumab.

A central feature of this case was the development of complete AV block in close temporal association with ICI exposure. New conduction abnormalities during ICI therapy should be regarded as an important warning sign for myocardial immune-related toxicity, particularly when accompanied by a substantial increase in cardiac troponin [11,12,13]. In this patient, the initial combination of intermittent chest discomfort, complete AV block, and markedly elevated hs-cTnT reasonably prompted evaluation for an acute coronary syndrome. However, coronary angiography demonstrated no obstructive coronary artery disease, while hs-cTnT subsequently increased from 2575 pg/mL on admission to a maximum of 11,327 pg/mL. The persistence and progression of myocardial injury after exclusion of an obstructive coronary cause represented an important opportunity to reconsider the initial diagnostic framework. However, troponin elevation was not specific for myocarditis in this complex clinical setting; contributions from renal dysfunction, bradycardia-related hemodynamic compromise, and concomitant abdominal disease cannot be excluded.

Temporary and subsequently permanent cardiac pacing addressed the clinically relevant conduction disturbance but did not explain the underlying myocardial injury. Repeat echocardiography during the index hospitalization continued to demonstrate preserved global left ventricular systolic function; after pacemaker implantation, apical rocking/dyskinesia was noted and considered potentially pacing-related. Thus, the severity of the clinical course was characterized predominantly by conduction disease, progressive myocardial injury, electrical instability, and circulatory deterioration rather than by documented severe global systolic dysfunction. In retrospect, the combination of recent dual ICI exposure, complete AV block, non-obstructive coronary arteries, and a pronounced dynamic hs-cTnT increase constituted a highly concerning constellation for ICI-associated myocarditis [4,11,12,13]. A wearable cardioverter-defibrillator was not considered because ICI-associated myocarditis had not been recognized during the hospitalization.

The marked CK elevation represents an additional relevant finding. Mechanistic studies support a T-cell-mediated basis for ICI-associated myocardial injury [14]. ICI-associated myocarditis may coexist with skeletal muscle immune-related toxicity, including myositis, and overlap syndromes may be associated with particularly severe clinical courses [6,15]. In the present case, however, the CK trajectory differed from that of hs-cTnT, with an initial decline from 8690 U/L followed by a secondary increase. Specific clinical features of myositis, such as muscle weakness or myalgia, cannot be reliably reconstructed from the available documentation, and no dedicated neuromuscular diagnostic work-up was performed. Concomitant ICI-associated myositis therefore remains possible but unconfirmed. In addition, chronic rosuvastatin therapy represents a potential alternative contributor to the CK elevation. Accordingly, the markedly elevated CK should not be interpreted as evidence of proven ICI-associated myositis. Whether skeletal muscle or diaphragm was sampled at autopsy could not be confirmed from the available pathology documentation.

An important competing consideration in this case was the retroperitoneal mass in the previous nephrectomy bed, which had been radiologically described as suspicious for a postoperative abscess with fat necrosis adjacent to the pancreas. The mass had already been visible on CT approximately three weeks before the terminal hospitalization, when the patient was clinically well, and no drainage or other local intervention had been performed. During the subsequent hemodynamic deterioration, an infectious contribution was therefore clinically plausible. Central venous blood cultures and urine cultures were obtained and ultimately remained negative; no tracheal secretion sample was collected. Empirical antimicrobial therapy was initiated.

The radiological suspicion of a postoperative abscess was acknowledged. However, septic shock was not established clinically during hospitalization. CRP was only moderately elevated, procalcitonin remained below the laboratory upper reference limit, no fever was reported, and the abdomen remained clinically unremarkable without documented upper abdominal pain. Although necrotizing pancreatitis was identified at autopsy in association with fistulation of the retroperitoneal abscess to the pancreas, lipase had been only minimally elevated during the hospitalization and subsequently normalized. Thus, neither the pancreatic involvement nor the retroperitoneal process had produced a clear clinical and laboratory constellation of clinically manifest acute pancreatitis or established septic shock before death. Nevertheless, the absence of characteristic symptoms, markedly elevated inflammatory markers, or positive microbiological cultures did not exclude severe localized infection or a contribution of the retroperitoneal abscess and pancreatic pathology to terminal circulatory failure.

Contemporary clinical documentation described a cardiogenic shock episode in the setting of complete AV block and prolonged bradycardia. However, repeat bedside echocardiography continued to show preserved global left ventricular systolic function and a completely collapsed inferior vena cava, while the concomitant retroperitoneal collection remained a relevant competing process. The terminal circulatory failure therefore cannot be assigned retrospectively to a single purely cardiogenic or distributive mechanism. The uncertain mechanism of the terminal shock should be distinguished from the post-mortem assessment of the cause of death. Autopsy showed pronounced diffuse lymphohistiocytic myocarditis involving both atria and ventricles and identified ICI-associated myocarditis as the cause of death. The abdominal pathology was classified as an aggravating condition rather than the primary cause of death. Preserved global left ventricular systolic function argues against severe global pump failure as the primary mechanism of shock but does not exclude clinically significant myocarditis with conduction abnormalities and ventricular arrhythmias.

Contemporary diagnostic pathways for suspected ICI-associated myocarditis incorporate cardiac biomarkers, electrocardiography, echocardiography, cardiac magnetic resonance imaging (CMR), and, in selected cases, endomyocardial biopsy. CMR can provide important diagnostic information, although characteristic findings may be absent, particularly early in the disease course. Therefore, a non-diagnostic CMR does not exclude ICI-associated myocarditis when clinical suspicion is high. Endomyocardial biopsy remains an important diagnostic modality when the diagnosis remains uncertain or when histopathological confirmation is clinically required [15,16,17,18,19].

In the present case, neither CMR nor myocarditis-directed endomyocardial biopsy was performed because ICI-associated myocarditis was not suspected during the hospitalization. The diagnosis can therefore be classified retrospectively as definite ICI-associated myocarditis on the basis of post-mortem histopathological confirmation. The clinical course was fulminant, with complete AV block, marked myocardial injury, progressive hemodynamic deterioration, ventricular tachycardia before cardiac arrest, recurrent ventricular tachycardia requiring defibrillation during resuscitation, and an initial documented arrest rhythm of pulseless electrical activity. Histopathological examination demonstrated pronounced diffuse lymphohistiocytic myocarditis involving both atria and ventricles, providing a pathological correlate for the severe electrical and clinical presentation. This pattern is consistent with mechanistic and histopathological studies demonstrating prominent T-cell and macrophage involvement in ICI-associated myocarditis [14,20]. The complete immunohistochemical panel, targeted examination of the atrioventricular node/His–Purkinje system, viral testing, and skeletal-muscle/diaphragmatic sampling could not be reliably retrieved from the available autopsy documentation and therefore represent limitations of this retrospective assessment.

Once ICI-associated myocarditis is suspected, current recommendations emphasize prompt interruption of ICI therapy and early initiation of high-dose corticosteroid treatment, with additional immunosuppressive strategies considered in severe or steroid-refractory disease [5,15,17,21,22]. In the present patient, corticosteroids or other myocarditis-directed immunosuppressive therapies were not administered because ICI-associated myocarditis was not recognized during the hospitalization. Whether earlier recognition and treatment would have altered the fatal course cannot be determined retrospectively. Nevertheless, observational evidence suggests that delays in appropriate immunosuppressive treatment may be associated with adverse outcomes in ICI-associated myocarditis.

The principal educational value of this case therefore lies not only in the post-mortem confirmation of ICI-associated myocarditis, but in the reconstruction of the clinical findings that preceded the diagnosis. In patients recently exposed to ICIs, particularly combination therapy, the occurrence of new high-grade conduction disease together with a marked dynamic increase in cardiac troponin should prompt immediate consideration of ICI-associated myocarditis, particularly once obstructive coronary artery disease has been excluded. Recognition of this constellation may facilitate earlier myocarditis-directed diagnostic evaluation and treatment [15,17,21].

## 4. Conclusions

ICI-associated myocarditis should be considered in patients receiving immune checkpoint inhibitors who develop new high-grade conduction abnormalities accompanied by a marked dynamic increase in cardiac troponin, particularly after obstructive coronary artery disease has been excluded. Severe ICI-associated myocarditis may manifest predominantly through conduction disease and electrical instability even in the absence of marked global left ventricular systolic dysfunction. In the present case, ICI-associated myocarditis was not recognized during the hospitalization, and consequently no myocarditis-directed diagnostic work-up or immunosuppressive treatment was initiated. The diagnosis was established only by post-mortem histopathological examination. Earlier recognition of this clinical constellation may facilitate timely myocarditis-directed diagnostic evaluation and treatment.

## Figures and Tables

**Table 1:** Clinical timeline from initiation of immune checkpoint inhibitor therapy to the fatal outcome and post-mortem diagnosis.

Date	Clinical Course	ECG/Cardiac Findings	Key Laboratory Findings	Diagnostic and Therapeutic Course
13 Jan 2026	Pre-ICI assessment	Preserved LV systolic function on TTE		Pre-treatment cardiac assessment
17 Feb 2026	First and only cycle of nivolumab + ipilimumab			Combined ICI therapy initiated
~3 Mar 2026	Onset of fatigue, reduced exercise tolerance, exertional dyspnea, and intermittent chest discomfort			Symptom onset approximately 14 days after ICI therapy
6 Mar 2026	Hospital admission	Complete third-degree AV block; ventricular rate ~35/min; QRS 140 ms; TTE with preserved LV systolic function and no pericardial effusion	hs-cTnT 2575 → 2857 pg/mL; CK 8690 → 7774 U/L; CK-MB 350 → 283 U/L; creatinine 2.86 mg/dL; eGFR 20 mL/min/1.73 m^2^	Working diagnoses: NSTEMI and complete AV block; ICI myocarditis not considered
7–8 Mar 2026	ICU monitoring	Persistent AV block; coronary angiography without obstructive CAD; temporary pacing VVI 70/min	7 Mar: hs-cTnT 3391 pg/mL; CK 7130 U/L; CK-MB 284 U/L. 8 Mar: CK 5011 U/L; CK-MB 221 U/L	Obstructive coronary cause excluded; temporary pacing continued
9 Mar 2026	Persistent conduction disorder	Permanent dual-chamber pacemaker implanted; DDD 50–150/min; subsequent interrogation with appropriate function and 71% ventricular pacing	hs-cTnT 8373 pg/mL; CK 5794–6444 U/L; CK-MB 298–354 U/L; NT-proBNP 20,538 pg/mL; CRP 14.5–15.5 mg/L; PCT 0.36–0.48 ng/mL; lipase 73 → 65 U/L	Permanent pacing; ICI myocarditis remained unrecognized
10 Mar 2026	Progressive hemodynamic deterioration; norepinephrine up to 0.5 µg/kg/min; abdomen clinically unremarkable; no fever reported	Effective paced rhythm ~70/min; repeat bedside TTE with preserved global LV function, apical rocking/dyskinesia potentially pacing-related, and completely collapsed IVC	hs-cTnT 11,327 → 10,326 pg/mL; CK 6707 → 6501 → 6206 U/L; CK-MB 389 → 371 → 368 U/L; NT-proBNP 29,534 pg/mL; CRP 13.5–14.4 mg/L; PCT 0.43–0.44 ng/mL; lipase 65 → 56 U/L	Microbiological sampling: central venous blood cultures and urine cultures; no tracheal secretion sample; all cultures ultimately negative; empirical piperacillin/tazobactam; contemporaneous documentation described the episode as cardiogenic shock in the context of prolonged bradycardia, although this classification cannot be confirmed retrospectively
11 Mar 2026	Progressive hypotension followed by cardiac arrest; prolonged CPR; resuscitation terminated 04:12	VT before arrest treated with amiodarone; initial arrest rhythm PEA at 03:28; recurrent VT during CPR requiring defibrillation	03:15: pH 7.228, lactate 52.0 mg/dL (5.8 mmol/L); 04:07: pH 7.031, lactate 98.6 mg/dL (10.9 mmol/L)	Advanced life support; repeated defibrillation for recurrent VT; ICI myocarditis remained unrecognized and no myocarditis-directed immunosuppression was given
Post mortem	Autopsy performed to clarify cause of death	Diffuse lymphohistiocytic myocarditis involving both atria and ventricles; heart weight 500 g		ICI myocarditis identified as cause of death; retroperitoneal abscess with pancreatic fistulation and necrotizing pancreatitis classified as aggravating pathology

Abbreviations: AV, atrioventricular; CAD, coronary artery disease; CK, creatine kinase; CK-MB, creatine kinase–myocardial band; CPR, cardiopulmonary resuscitation; CRP, C-reactive protein; DDD and VVI, pacemaker modes; hs-cTnT, high-sensitivity cardiac troponin T; ICI, immune checkpoint inhibitor; LV, left ventricular; NSTEMI, non-ST-elevation myocardial infarction; NT-proBNP, N-terminal pro-B-type natriuretic peptide; PCT, procalcitonin; PEA, pulseless electrical activity; TTE, transthoracic echocardiography; VT, ventricular tachycardia.

## Data Availability

Data published in this case report are available from the corresponding author upon reasonable request.

## Abbreviations

The following abbreviations are used in this manuscript:

CK	Creatine kinase
CK-MB	Creatine kinase–myocardial band
CMR	Cardiac magnetic resonance imaging
CRP	C-reactive protein
CT	Computed tomography
CTLA-4	Cytotoxic T-lymphocyte-associated protein 4
ECG	Electrocardiogram
hs-cTnT	High-sensitivity cardiac troponin T
ICI	Immune checkpoint inhibitor
ICU	Intensive care unit
irAE	Immune-related adverse event
NT-proBNP	N-terminal pro-B-type natriuretic peptide
PD-1	Programmed cell death protein 1
PD-L1	Programmed death-ligand 1
UICC	Union for International Cancer Control
